# Integrating preoperative multiregion radiomic features with clinical data to predict atrial fibrillation recurrence after radiofrequency ablation

**DOI:** 10.3389/fcvm.2026.1923774

**Published:** 2026-08-26

**Authors:** Hui Yan, Xiao-Le Li, Jun-Hao Mei, Lei Zhou, Shu-Hao Guo, Li-Xiang Xie, Xiao-Yun Zhu, Chun-Feng Hu, Chang-Jie Pan, Hui Xue

**Affiliations:** 1 Department of Radiology, Jintan Affiliated Hospital of Jiangsu University, Changzhou, China; 2 Department of Radiology, Xuzhou Central Hospital, Affiliated Hospital of Southeast University, Xuzhou, China; 3 Department of Interventional Radiology, Zhongda Affiliated Hospital of Southeast University, Nanjing, China; 4 Department of Cardiology, Jintan Affiliated Hospital of Jiangsu University, Changzhou, China; 5 Department of Radiology, Affiliated Hospital of Xuzhou Medical University, Xuzhou, China; 6 Department of Radiology, Affiliated Changzhou No.2 People’s Hospital of Nanjing Medical University, Changzhou, China

**Keywords:** atrial fibrillation, machine learning, radiofrequency ablation, radiomics, recurrence prediction

## Abstract

**Objective:**

To develop and validate a prediction framework integrating clinical data, left atrium and pulmonary vein morphology, and radiomic features to identify patients at high risk of atrial fibrillation (AF) recurrence after radiofrequency ablation (RFA).

**Methods:**

Patients with AF who underwent RFA at three centers between August 2018 and October 2024 were retrospectively screened. Patients from two centers were divided into training and internal validation cohorts, and patients from the third center formed the external validation cohort. Clinical and CTA-derived morphological variables and radiomic features from the left atrium and epicardial adipose tissue were analyzed. Clinical, radiomic, and fusion models were evaluated using five machine-learning algorithms. Training performance was estimated from five-fold out-of-fold predictions. Discrimination, calibration, clinical utility, pairwise AUROC differences, and model contributions were assessed using ROC analysis, calibration plots, decision curve analysis, DeLong tests, and SHAP.

**Results:**

Of 877 patients, 449 formed the training cohort, 192 the internal validation cohort, and 236 the external validation cohort; recurrence occurred in 23.6%, 30.2%, and 35.2%, respectively. The optimal clinical GBDT, radiomic RF, and fusion RF models achieved AUROCs of 0.739, 0.922, and 0.947 in the training cohort; 0.726, 0.837, and 0.839 in internal validation; and 0.722, 0.837, and 0.848 in external validation. Radiomic and fusion models outperformed the clinical model in both validation cohorts, whereas fusion did not significantly outperform radiomic (DeLong *P* = 0.95 internally and *P* = 0.43 externally). Decision curves showed net benefit across relevant threshold ranges, while calibration varied across models and cohorts.

**Conclusion:**

Radiomic models substantially improved post-RFA recurrence discrimination over the clinical model. The fusion model achieved the numerically highest external AUROC, but did not significantly outperform radiomic alone in either validation cohort. These findings support radiomic-based risk stratification while underscoring the need for prospective calibration and validation.

## 1 Introduction

Atrial fibrillation (AF), the most common clinical arrhythmia, is associated with a substantially increased risk of heart failure, stroke, and myocardial infarction and thus places a heavy health and economic burden on patients and society (1). More than 33 million people worldwide are living with AF (2), and this number continues to increase each year. AF has therefore become an increasingly pressing public health issue.

AF can be treated with a variety of approaches, including medication and surgical intervention. Another treatment approach is radiofrequency ablation (RFA), which is a minimally invasive strategy that offers rapid symptom relief and has been endorsed by multiple groups as the preferred nonpharmacological treatment for AF (3). However, studies have shown that the recurrence rate of AF within 1 year after RFA can be as high as 20% to 50% (4–6). This high recurrence rate underscores the need for clinical screening of patients at high risk of recurrence to enable timely postoperative adjuvant therapy and proactive follow-up strategies.

In recent years, numerous risk assessment tools for AF recurrence have emerged. For instance, previous studies have shown that conventional clinical parameters such as AF type, CHA₂DS₂-VASc score, and N-terminal pro-B-type natriuretic peptide (NT-proBNP) level may be associated with AF recurrence after RFA (3, 5, 7–9). However, the predictive performance of these parameters has been inconsistent and is often inadequate for individualized risk stratification. Imaging features such as left atrium size and the morphology of the pulmonary vein ostia have also demonstrated some predictive value; however, because of substantial interindividual variability, the predictive stability of these features is limited (10, 11). Artificial intelligence (AI) tools such as machine learning, deep learning, and radiomic have shown substantial potential in postoperative prognostic assessment after RFA (12, 13), but these approaches often depend on large volumes of high-dimensional data and remain limited in their ability to integrate multimodal information. A more interpretable integrated model based on multimodal clinical data is needed to identify patients at high risk of recurrence at an earlier stage and guide individualized treatment. This may help reduce recurrence rates and improve both therapeutic efficacy and quality of life (14–16).

The goal of this study, therefore, was to develop and validate a robust risk model for the recurrence of AF after RFA using baseline clinical data, laboratory indicators, morphological parameters of the left atrium and pulmonary vein, and radiomic features.

## 2 Methods

### 2.1 Study design and participating cohorts

We retrospectively screened consecutive patients with AF who underwent RFA between August 1, 2018, and October 31, 2024, at one of three tertiary centers in China. Indications for ablation and definitions of recurrence were based on the 2024 European Heart Rhythm Association/Heart Rhythm Society/Asia Pacific Heart Rhythm Society/Latin American Heart Rhythm Society expert consensus statement (3). Eligible patients underwent substrate-guided RFA performed by experienced electrophysiologists, had preprocedural left atrial–pulmonary vein computed tomography angiography (CTA) of sufficient quality for analysis, had not undergone electrical or pharmacological cardioversion within 3 months before ablation, and completed scheduled rhythm monitoring during follow-up. Patients were excluded from the analysis if they had structural heart disease (including congenital heart disease, cardiomyopathy, or severe valvular disease), had severe hepatic or renal dysfunction, had experienced cerebrovascular or cardiovascular events, had an acute infection, had undergone surgery within the preceding 3 months before RFA, had undergone a previous electrophysiological intervention or AF ablation, or had incomplete clinical or imaging data.

**Figure 1:**
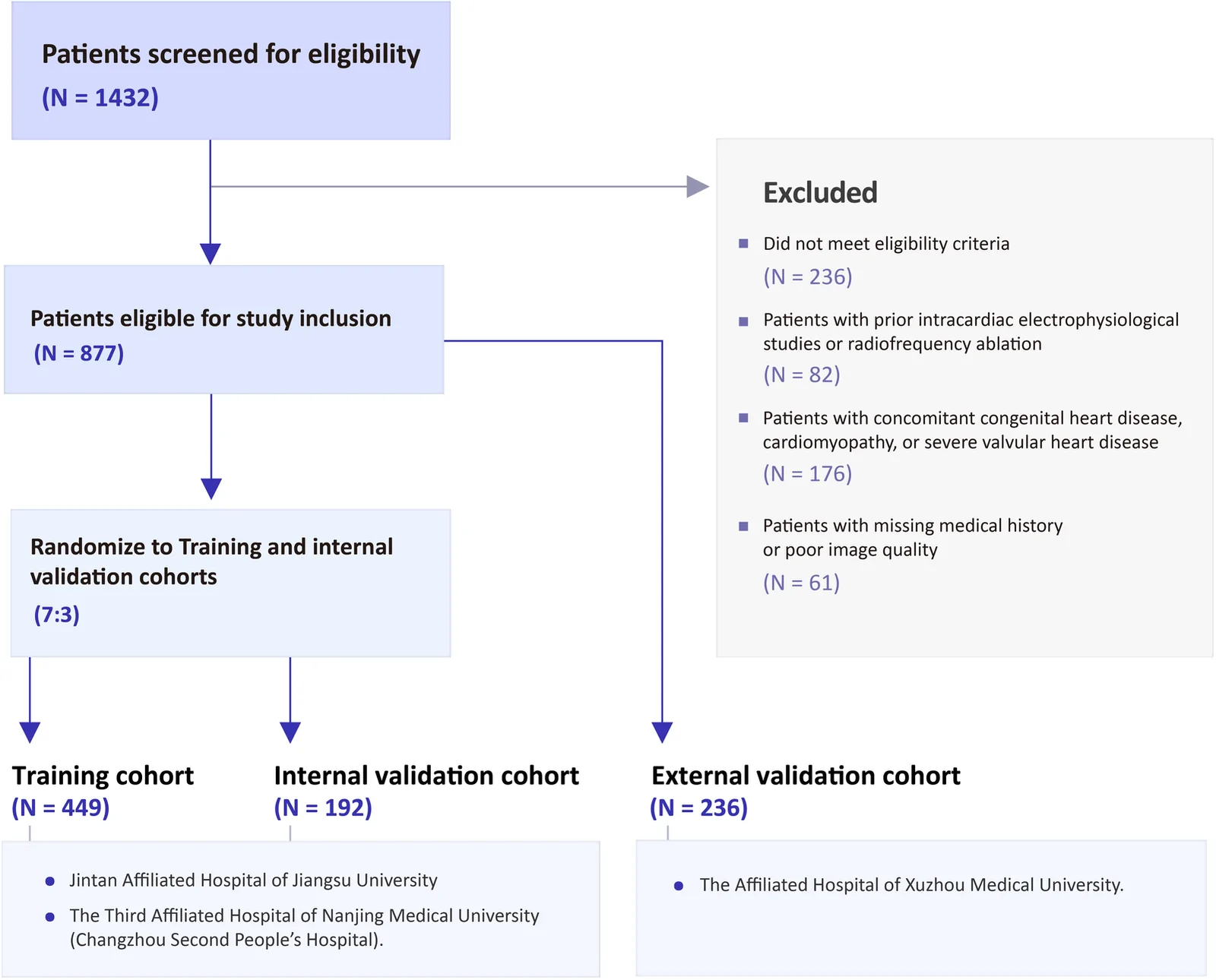
Flow chart of study design.

Patients from two of the three centers were randomly allocated by a computer to the training cohort or the internal validation cohort in a 7:3 ratio. Patients from the remaining center were assigned to the external validation cohort (Figure 1).

This study was approved by the Ethics Committee of authors' respective institutions [Ethics Approval No. 2025-KY-002; XYFY2023-KL225-01; (2022)KY120-01], and informed consent was waived for all enrolled patients. The requirement for informed consent was waived for all study patients. The study followed the STROBE guidelines (17) and the TRIPOD + AI statement (18).

### 2.2 Treatments

All patients received oral anticoagulation on the day of ablation. Patients at high thromboembolic risk, defined by a CHA_2_DS_2_-VASc score of at least 2 in men or 3 in women, continued anticoagulation for a minimum of 3 months after RFA and, when clinically indicated, for longer than 12 months. With the exception of amiodarone, antiarrhythmic drugs were discontinued for at least five half-lives before the procedure (19).

RFA was performed using a standard transseptal approach with the patient under local anaesthesia. Electroanatomical mapping of the left atrium was undertaken with the CARTO system, followed by circumferential isolation of the pulmonary veins guided by a circular mapping catheter. Additional substrate modification was performed at the operator's discretion when clinically indicated. Electrical cardioversion was used if sinus rhythm was not restored after ablation. Procedural success was confirmed by demonstration of pulmonary vein isolation and bidirectional conduction block after a 20 -min waiting period. Detailed procedural protocols are provided in the Supplementary Material.

### 2.3 Data collection

Preprocedural contrast-enhanced CTA of the left atrium and pulmonary veins was performed using multidetector CT scanners (Somatom FORCE, Siemens Healthineers, Germany; or Revolution CT, GE Healthcare, USA). Scans were acquired using electrocardiogram (ECG)-gated or nongated protocols according to cardiac rhythm status. Images were reconstructed at end-diastole with a slice thickness of 0.75 mm and were then transferred to a dedicated workstation for multiplanar and three-dimensional analyses. Detailed acquisition parameters are provided in the Supplementary Material.

Baseline clinical variables were collected before RFA and included demographic characteristics, AF subtype, cardiovascular risk factors, laboratory measurements (including triglyceride-glucose index, glucose level, hemoglobin level, renal function marker levels, lipid profiles, gamma-glutamyl transferase level, uric acid level, and NT-proBNP level), and established risk scores (CHA_2_DS_2_-VASc and HAS-BLED).

Morphological parameters of the left atrium and pulmonary veins were derived from CTA multiplanar reconstructions. The left atrial transverse diameter (LAD) and vertebral body transverse diameter (VD) were measured on the axial plane through the right inferior pulmonary vein ostium, and the left atrio-vertebral diameter ratio was calculated as LAD/VD. The superior-inferior diameter (LA1) and anteroposterior diameter (LA2) were measured on a sagittal reconstruction aligned with the left atrial long axis; together with LAD, these represented three approximately orthogonal diameters. Left atrial volume was estimated using a triaxial ellipsoid approximation (π/6 × LAD × LA1 × LA2), and left atrial sphericity was calculated as the equal-volume sphere diameter divided by the largest of LAD, LA1, and LA2. Pulmonary vein ostial maximal and minimal diameters and cross-sectional areas were also measured. Detailed definitions are provided in Supplementary Table S1 and Figure S1.

### 2.4 Follow-up

Patients underwent 24-h Holter monitoring at 1, 3, 6, 9, and 12 months after RFA during a prespecified 12-month follow-up period. A 90-day blanking period was applied after ablation. Episodes of AF, atrial flutter, or atrial tachycardia occurring within the first 90 days, including those detected at the 1-month assessment, were classified as early post-ablation atrial arrhythmias and were not included in the primary endpoint. The 3-month assessment served as the boundary of the blanking period; only episodes documented after completion of the 90-day blanking period were counted as recurrence. The primary endpoint was defined as any objectively documented episode of AF, atrial flutter, or atrial tachycardia lasting more than 30 s between the end of the blanking period and the 12-month assessment (3, 20, 21). For patients who did not return to a participating centre, rhythm status was ascertained by telephone review of ECG or Holter reports obtained at other hospitals; symptoms reported by telephone without objective rhythm documentation were not sufficient to establish recurrence.

### 2.5 Model training and performance

Two radiologists followed a standard operating procedure (SOP) to manually delineate the left atrial and epicardial adipose tissue ROIs on preprocedural CTA images using 3D Slicer (National Institutes of Health, Bethesda, MD, USA) (Supplementary Figure S2). Discrepancies were resolved by consensus (22). Fifty examinations were randomly selected for reproducibility assessment, and one reader repeated segmentation after 4 weeks. Interobserver and intraobserver agreement were assessed using absolute-agreement ICCs; only features with both ICCs ≥0.80 were retained. Images and masks were resampled to isotropic 1 × 1 × 1 mm^3^ voxels using B-spline and nearest-neighbour interpolation, respectively. Image-level intensity normalization was not applied, and CT intensities were discretized using a fixed bin width of 25 HU. First-order, shape, and texture features were extracted from original images using PyRadiomic version 3.0.1; LoG and wavelet filters were not applied. Features were *Z*-score standardized using training-cohort parameters and transformed identically in validation cohorts.

**Figure 2:**
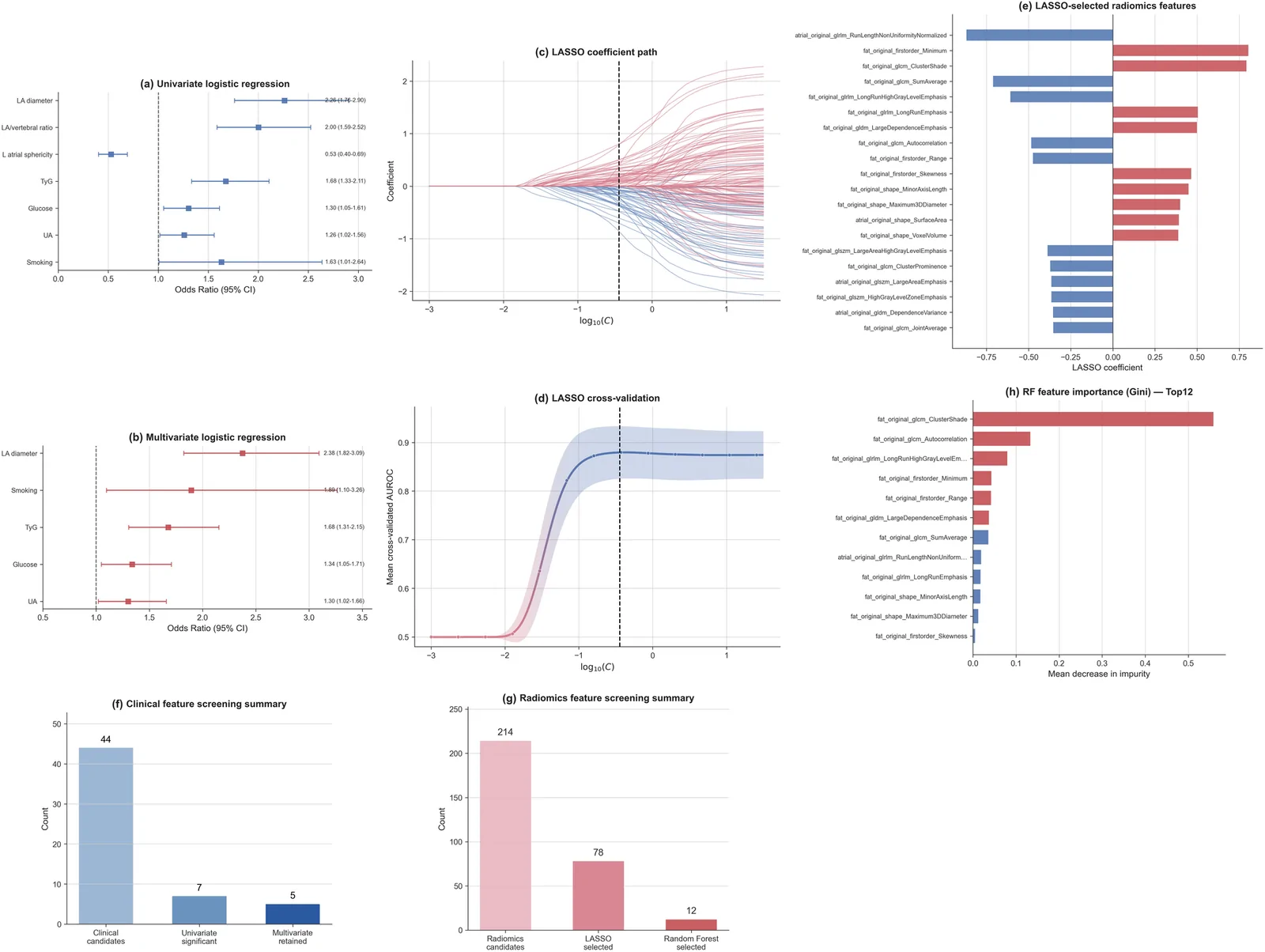
Summary of the clinical and radiomic feature-selection workflow. **(a,b)** Forest plots of univariable and multivariable logistic regression for clinical features. **(c)** LASSO coefficient path plot. **(d)** Cross-validation curve for LASSO regression. **(e)** Coefficients of LASSO-selected radiomic features. **(f,g)** Clinical and radiomic feature-screening summaries. **(h)** Final 12 radiomic features ranked by random-forest Gini importance. All feature-selection procedures were performed in the training cohort.

Feature selection was conducted exclusively in the training cohort. Among 44 candidate clinical and morphological variables, seven met the univariable logistic-regression criterion (two-sided *P* < 0.05); continuous variables were standardized before regression, so odds ratios were expressed per 1-SD increase. Five variables: left atrial diameter, triglyceride–glucose index, smoking history, glucose, and uric acid remained significant in multivariable analysis. For radiomic, variance and correlation filtering were followed by LASSO regression, which retained 78 features. Random-forest Gini importance was then used to select the 12 highest-ranking features (Figure 2).

Three model families were evaluated: a five-variable clinical model, a 12-feature radiomic model, and a 17-feature fusion model. Each feature set was assessed using logistic regression, support vector machine, random forest, gradient-boosting decision tree, and K-nearest neighbours, yielding 15 sub-models. Training performance was estimated from pooled out-of-fold probabilities generated by five-fold stratified cross-validation. Each final algorithm was then refitted on the complete training cohort and applied unchanged to the internal and external validation cohorts. Validation cohorts were not used for feature selection, hyperparameter tuning, or model fitting.

**Figure 3:**
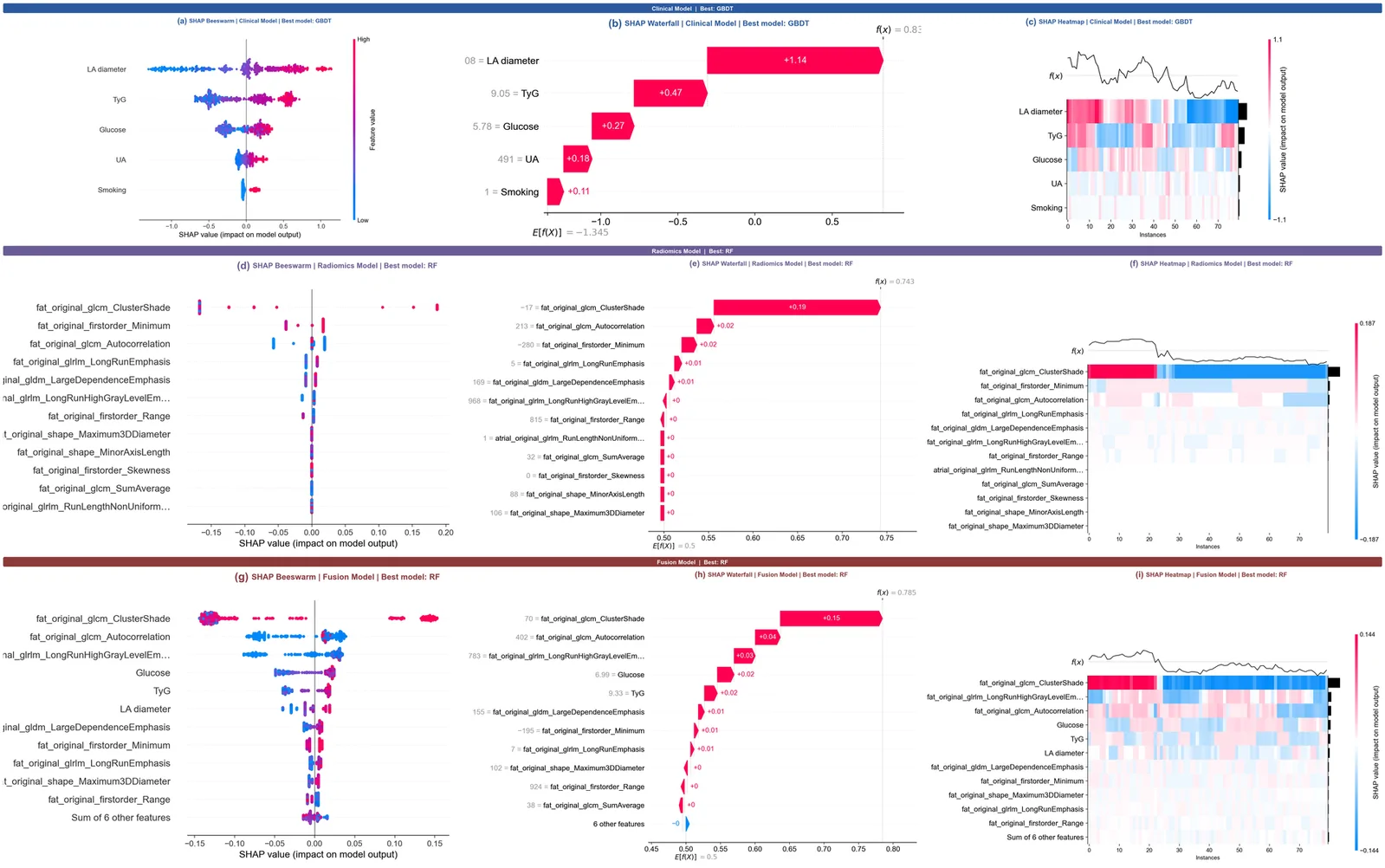
SHAP-based interpretation of the optimal clinical, radiomic, and fusion models. Beeswarm plots summarize global feature contributions for the clinical **(a)**, radiomic **(d)**, and fusion **(g)** models. Waterfall plots illustrate representative individual predictions **(b,e,h)**, and heatmaps display feature-contribution patterns across selected patients **(c,f,i)**.

All 15 sub-models were reported. For descriptive comparison and SHAP visualization, the algorithm with the highest external-validation AUROC within each feature family was designated the best-performing model: GBDT for the clinical model and RF for the radiomic and fusion models. SHAP beeswarm plots summarized global feature contributions, waterfall plots illustrated representative individual predictions, and heatmaps displayed contribution patterns across 80 randomly selected patients (Figure 3). SHAP values were interpreted as contributions to model output, not as evidence of biological causation (23).

### 2.6 Statistical analysis

All analyses were conducted using R version 4.4.3. Continuous variables are reported as median (IQR) and categorical variables as *n* (%). Between-group comparisons used Student’s *t* test or the Mann–Whitney *U*-test for continuous variables and the *χ*^2^ test or Fisher's exact test for categorical variables, as appropriate. AUROCs are reported with 95% CIs estimated from 1,000 bootstrap resamples. Calibration was assessed using calibration curves, slopes, and intercepts; clinical utility was evaluated by decision curve analysis. Pairwise AUROC differences were tested using the paired DeLong method. Threshold-dependent metrics were descriptive and calculated at the cohort-specific threshold maximizing the Youden index. All tests were two-sided, with *P* < 0.05 denoting statistical significance.

## 3 Results

### 3.1 Cohort characteristics

Of the 1,432 patients initially screened for study eligibility, 555 were excluded based on the exclusion criteria (Figure 1). The remaining 877 patients were divided into the training cohort (*n* = 449), the internal validation cohort (*n* = 192), and the external validation cohort (*n* = 236). Baseline characteristics were assessed across all three cohorts (Table 1). The cohorts exhibited significant differences in low-density lipoprotein level (*p* < 0.001), left atrial diameter (*p* < 0.001), left atrio-vertebral diameter ratio (*p* < 0.001), left superior pulmonary vein maximal diameter (*p* = 0.006), and left atrial sphericity (*p* = 0.002). The AF recurrence rate after RFA was 23.6% in the training cohort, 30.2% in the internal validation cohort, and 35.2% in the external validation cohort.

**Table 1:** Baseline characteristics of study patients.

Variable	Cohort			*p* value*
	Training (*n* = 449)	Internal (*n* = 192)	External (*n* = 236)	
Recurrence rate	106 (23.6)	58 (30.2)	83 (35.2)	0.005
Age (y)	62.00 (52.00, 69.00)	60.00 (49.00, 67.00)	62.00 (53.00, 67.00)	0.257
Sex				0.896
Male	295 (65.7)	124 (64.6)	151 (64.0)	
Female	154 (34.3)	68 (35.4)	85 (36.0)	
Triglyceride level, mmol/L	1.47 (0.98, 1.96)	1.46 (1.08, 1.99)	1.52 (1.09, 1.99)	0.663
Triglyceride-glucose index	8.73 (8.33, 9.07)	8.70 (8.35, 9.09)	8.75 (8.46, 9.17)	0.287
Heart rate, bpm	78.00 (67.00, 92.00)	75.50 (66.75, 86.00)	80.00 (68.75, 92.00)	0.053
Height, cm	169.00 (160.00, 177.00)	167.50 (158.75, 176.00)	169.50 (159.00, 176.00)	0.442
Weight, kg	73.00 (63.00, 82.00)	70.50 (60.00, 81.00)	73.00 (65.00, 80.25)	0.394
Hemoglobin level, g/L	143.00 (132.00, 155.00)	144.50 (132.75, 155.00)	141.00 (130.00, 155.00)	0.546
Urea level, mmol/L	5.59 (4.43, 6.92)	5.75 (4.60, 7.16)	5.50 (4.64, 6.55)	0.591
Serum creatinine level, µmmol/L	68.00 (58.00, 77.00)	68.00 (60.00, 79.00)	65.50 (59.00, 75.00)	0.427
Uric acid level, µmmol/L	324.00 (269.00, 399.00)	328.50 (272.75, 391.50)	319.00 (269.75, 377.25)	0.468
Gamma-glutamyl transferase level, U/L	30.00 (17.00, 52.00)	31.00 (14.75, 51.00)	27.50 (17.00, 43.00)	0.740
Total cholesterol level, mmol/L	4.23 (3.65, 4.83)	4.21 (3.51, 4.80)	4.46 (3.74, 4.99)	0.066
Glucose level, mmol/L	5.30 (4.52, 6.59)	5.08 (4.32, 6.21)	5.39 (4.82, 6.17)	0.076
High-density lipoprotein level, mmol/L	1.07 (0.91, 1.25)	1.08 (0.92, 1.29)	1.08 (0.93, 1.28)	0.547
Low-density lipoprotein level, mmol/L	2.32 (1.78, 2.60)	2.32 (1.51, 2.39)	2.53 (2.02, 3.00)	<0.001
N-terminal pro-brain natriuretic peptide level, pg/mL	549.79 (156.59, 1,004.81)	608.28 (184.61, 997.72)	466.45 (147.38, 934.00)	0.923
CHA2DS2-VASc score	2.00 (1.00, 3.00)	2.00 (1.00, 3.00)	2.00 (1.00, 3.00)	0.798
HAS-BLED score	1.00 (0.00, 2.00)	1.00 (1.00, 2.00)	1.00 (0.00, 2.00)	0.661
Left atrial diameter, cm	3.75 (3.28, 4.20)	3.75 (3.37, 4.11)	4.02 (3.49, 4.61)	<0.001
Vertebral body diameter, cm	1.62 (1.50, 1.75)	1.64 (1.50, 1.75)	1.61 (1.49, 1.75)	0.721
Left atrio-vertebral diameter ratio	2.30 (2.01, 2.67)	2.24 (2.00, 2.59)	2.50 (2.13, 2.95)	<0.001
Left superior pulmonary vein				
Maximal diameter, cm	2.33 (1.96, 2.69)	2.32 (1.90, 2.61)	2.41 (2.05, 2.88)	0.006
Minimal diameter, cm	1.41 (1.22, 1.72)	1.42 (1.22, 1.69)	1.42 (1.22, 1.73)	0.642
Cross-sectional area, cm^2^	2.32 (1.59, 2.95)	2.19 (1.46, 2.87)	2.36 (1.66, 3.09)	0.237
Left inferior pulmonary vein				
Maximal diameter, cm	1.49 (1.31, 1.71)	1.50 (1.33, 1.68)	1.55 (1.34, 1.74)	0.140
Minimal diameter, cm	1.04 (0.84, 1.21)	1.02 (0.86, 1.18)	1.02 (0.82, 1.19)	0.756
Cross-sectional area, cm^2^	1.26 (0.91, 1.62)	1.17 (0.86, 1.58)	1.32 (0.91, 1.63)	0.133
Right superior pulmonary vein				
Maximal diameter, cm	1.94 (1.65, 2.22)	1.92 (1.65, 2.18)	1.98 (1.72, 2.27)	0.155
Minimal diameter, cm	1.56 (1.32, 1.83)	1.60 (1.31, 1.86)	1.60 (1.36, 1.87)	0.672
Cross-sectional area, cm^2^	2.82 (2.17, 3.57)	2.82 (2.16, 3.73)	3.03 (2.38, 3.70)	0.068
Right inferior pulmonary vein				
Maximal diameter, cm	1.44 (1.12, 1.74)	1.38 (1.13, 1.67)	1.46 (1.20, 1.70)	0.455
Minimal diameter, cm	1.25 (1.05, 1.47)	1.23 (0.99, 1.50)	1.25 (1.04, 1.51)	0.687
Cross-sectional area, cm^2^	1.57 (1.09, 2.16)	1.57 (1.04, 2.02)	1.58 (1.07, 2.26)	0.561
Left atrium				
Maximal diameter, cm	6.25 (5.42, 7.02)	6.32 (5.40, 6.93)	6.38 (5.59, 7.08)	0.790
Minimal diameter, cm	4.61 (4.07, 5.03)	4.57 (4.07, 5.03)	4.63 (4.02, 5.21)	0.676
Volume, cm^3^	58.37 (41.87, 73.99)	57.32 (41.44, 75.98)	60.09 (44.19, 75.73)	0.457
Sphericity	0.63 (0.54, 0.72)	0.64 (0.57, 0.71)	0.60 (0.51, 0.70)	0.002
Atrial fibrillation type				0.075
Paroxysmal	275 (61.2)	105 (54.7)	125 (53.0)	
Persistent	174 (38.8)	87 (45.3)	111 (47.0)	
History of hypertension				0.764
No	232 (51.7)	105 (54.7)	126 (53.4)	
Yes	217 (48.3)	87 (45.3)	110 (46.6)	
History of diabetes				0.133
No	373 (83.1)	154 (80.2)	206 (87.3)	
Yes	76 (16.9)	38 (19.8)	30 (12.7)	
History of smoking				0.514
No	338 (75.3)	150 (78.1)	173 (73.3)	
Yes	111 (24.7)	42 (21.9)	63 (26.7)	
History of alcohol use				0.742
No	367 (81.7)	158 (82.3)	188 (79.7)	
Yes	82 (18.3)	34 (17.7)	48 (20.3)	

Data are *n* (%) or median (IQR). NT-proBNP, N-terminal pro-brain natriuretic peptide.

* *p* values for training cohort vs. internal cohort vs. external cohort.

Recurrence-stratified baseline characteristics are provided in Supplementary Table S3. Left atrial diameter and the left atrio-vertebral diameter ratio differed between recurrence groups in all three cohorts. The triglyceride–glucose index and glucose differed in the training and external validation cohorts, whereas validation-cohort comparisons were descriptive and did not inform feature selection or model optimization.

### 3.2 Model construction and performance

In the training cohort, seven of 44 candidate clinical and morphological variables met the univariable criterion, and five: left atrial diameter, triglyceride–glucose index, smoking history, glucose, and uric acid were retained in multivariable analysis. Of 214 candidate radiomic features, 78 remained after variance/correlation filtering and LASSO regression; the 12 highest-ranking features by random-forest Gini importance formed the final radiomic subset (Figure 2). The resulting clinical, radiomic, and fusion inputs comprised 5, 12, and 17 features, respectively.

**Figure 4:**
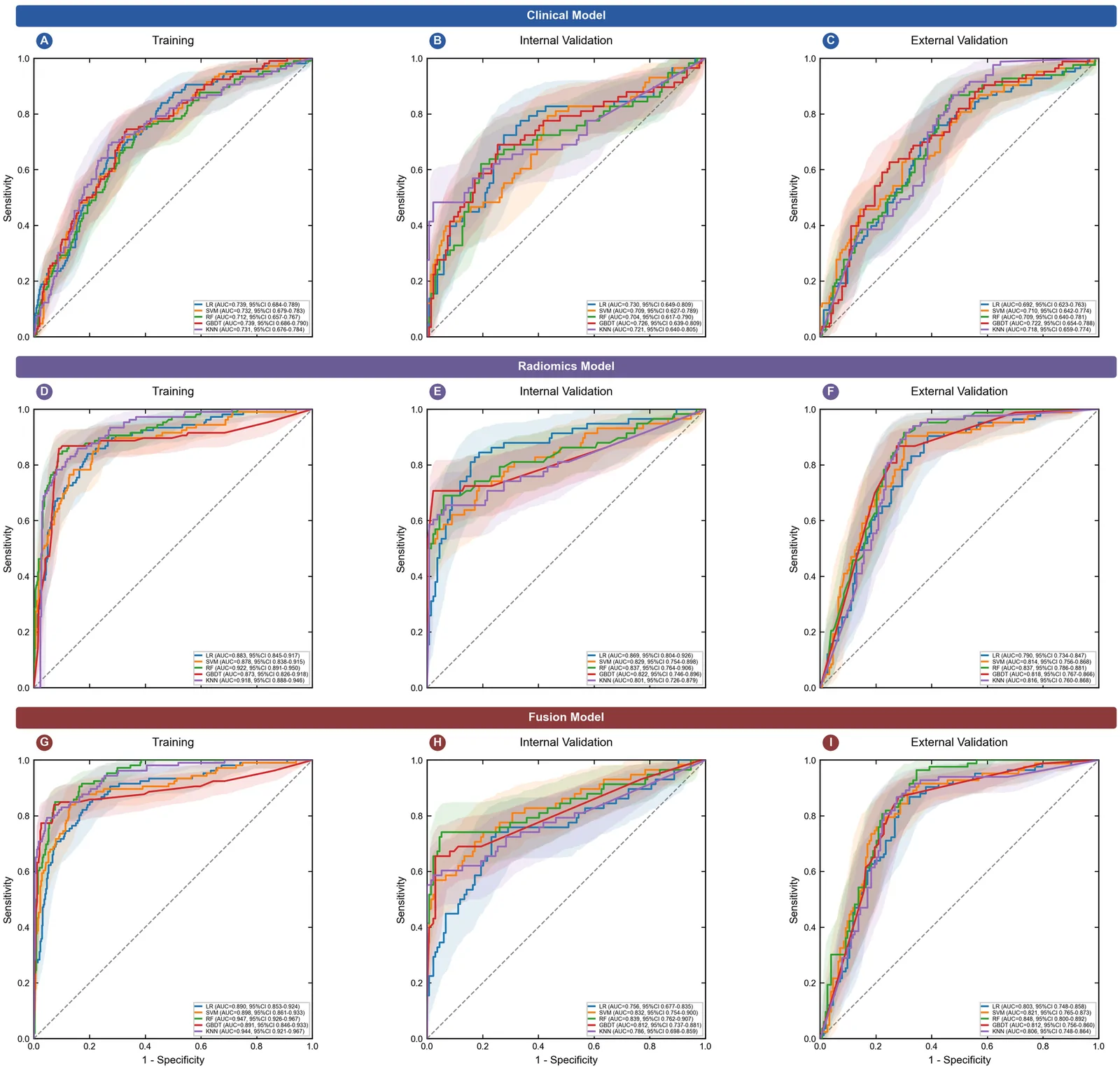
Discrimination performance of all sub-models. Receiver operating characteristic curves illustrate the predictive performance of the clinical **(A–C)**, radiomic **(D–F)**, and fusion **(G–I)** models in the training, internal validation, and external validation cohorts, respectively. Shaded areas represent 95% confidence intervals.

All five algorithms were evaluated within each feature family (Figure 4 and Supplementary Table S5). Based on external-validation AUROC, GBDT was the best-performing clinical algorithm, whereas RF was the best-performing radiomic and fusion algorithm. These designations were used for cross-family comparison and SHAP analysis; complete results for all 15 sub-models are provided in Supplementary Table S5.

**Figure 5:**
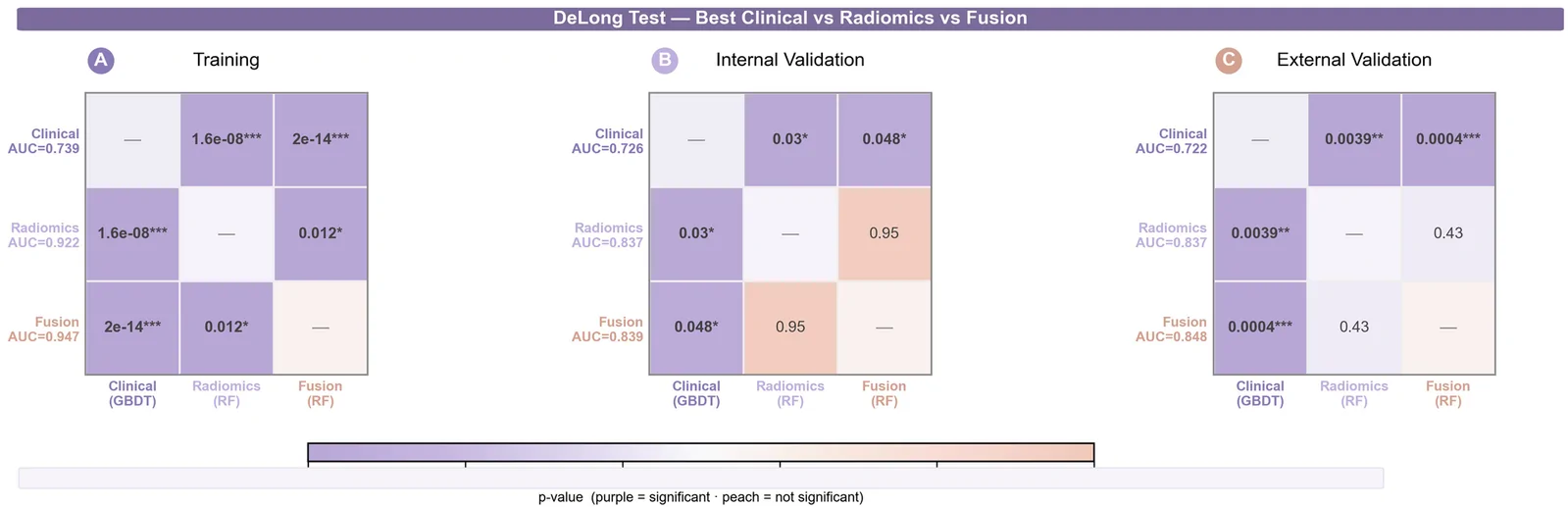
Pairwise comparison of the optimal clinical, radiomic, and fusion models. DeLong tests compare the AUROCs of the optimal clinical, radiomic, and fusion models in the training **(a)**, internal validation **(b)**, and external validation **(c)** cohorts.

**Figure 6:**
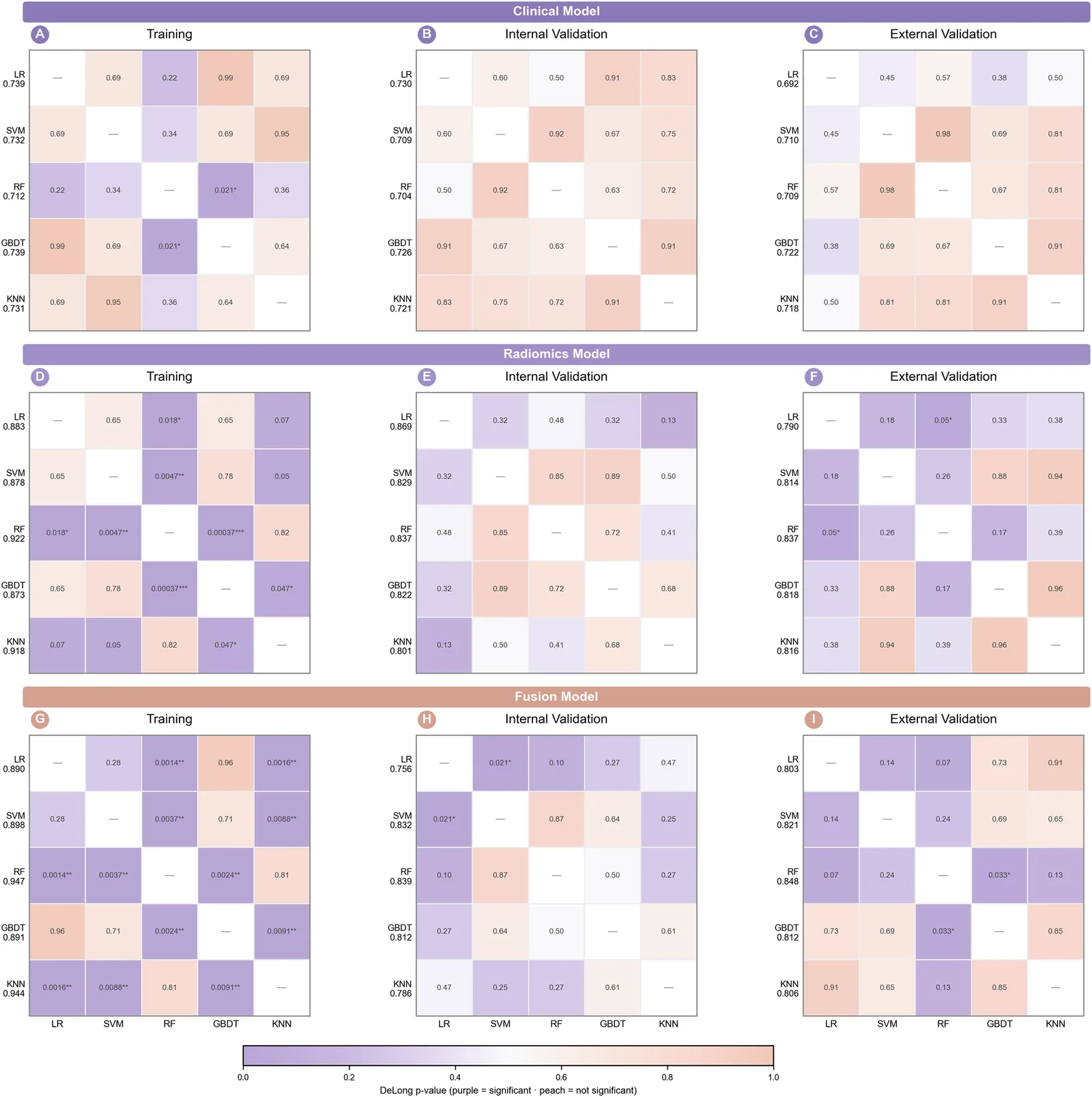
Pairwise comparison of discrimination among submodels. Heatmaps present DeLong test *P* values for pairwise AUROC comparisons among the five algorithms within the clinical **(A–C)**, radiomic **(D–F)**, and fusion **(G–I)** models across the training, internal validation, and external validation cohorts, respectively.

The clinical GBDT model achieved AUROCs of 0.739 (95% CI 0.686–0.790), 0.726 (0.639–0.809), and 0.722 (0.654–0.788) in the training, internal validation, and external validation cohorts, respectively. Corresponding AUROCs were 0.922 (0.891–0.950), 0.837 (0.764–0.906), and 0.837 (0.786–0.881) for the radiomic RF model, and 0.947 (0.926–0.967), 0.839 (0.762–0.907), and 0.848 (0.800–0.892) for the fusion RF model (Figure 4 and Supplementary Table S5). The fusion model outperformed the radiomic model in training (DeLong *P* = 0.012), but not in internal (*P* = 0.95) or external validation (*P* = 0.43). Both radiomic and fusion models outperformed the clinical model in internal validation (*P* = 0.03 and *P* = 0.048) and external validation (*P* = 0.0039 and *P* = 0.0004), respectively (Figure 5). Most pairwise differences among algorithms within each model family were not significant in the validation cohorts (Figure 6).

**Figure 7:**
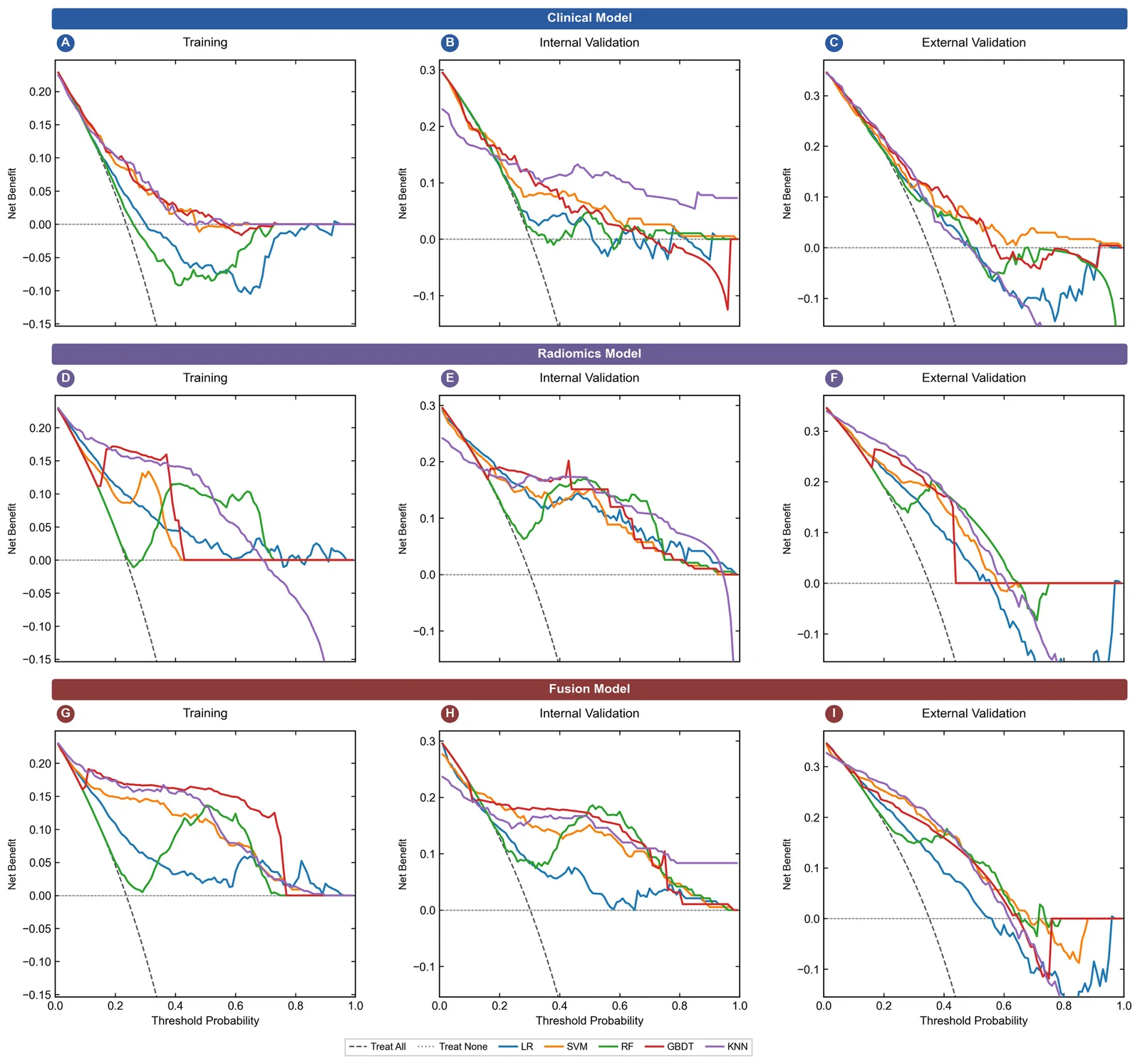
Clinical utility of all sub-models. Decision curve analysis evaluates net benefit across threshold probabilities for the clinical **(A–C)**, radiomic **(D–F)**, and fusion **(G–I)** models in the training, internal validation, and external validation cohorts, respectively.

**Figure 8:**
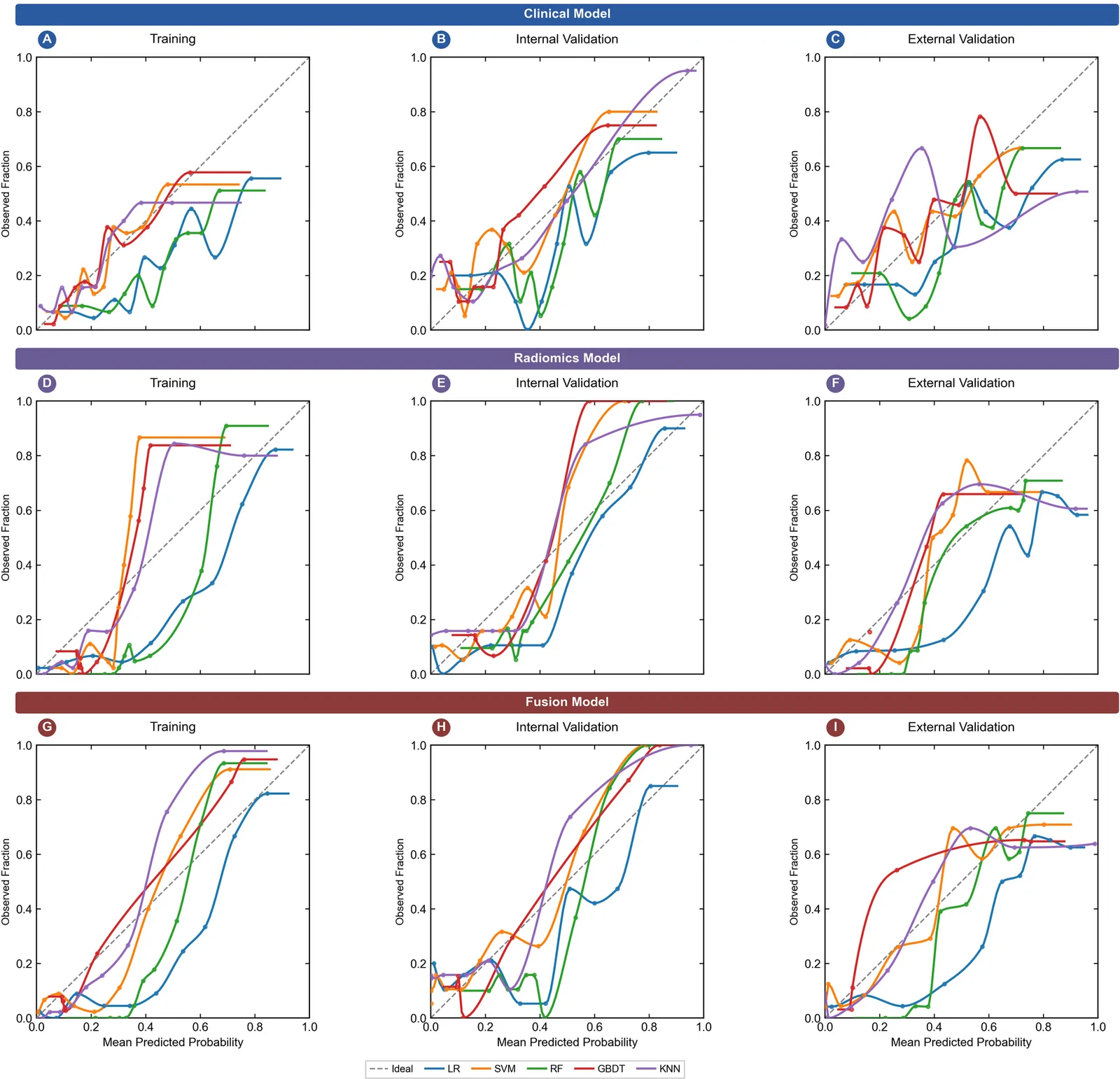
Calibration performance of all submodels. Calibration plots assess agreement between predicted probabilities and observed AF recurrence in the clinical **(A–C)**, radiomic **(D–F)**, and fusion **(G–I)** models across the training, internal validation, and external validation cohorts, respectively. The diagonal line represents perfect calibration.

Calibration and net benefit varied by algorithm and cohort (Figures 7, 8). In external validation, calibration slopes/intercepts were 0.806/−0.032 for the clinical GBDT model, 1.687/−0.841 for the radiomic RF model, and 2.021/−0.870 for the fusion RF model. At cohort-specific Youden thresholds, external-validation sensitivity/specificity was 0.627/0.752, 0.916/0.699, and 0.964/0.654, respectively (Supplementary Table S5). Decision curves showed positive net benefit over clinically relevant threshold ranges, although the magnitude and range varied across models.

Scanner-stratified radiomic RF AUROCs were 0.930 and 0.908 in the Siemens and GE training subsets, 0.845 and 0.826 in internal validation, and 0.829 and 0.842 in external validation, respectively (Supplementary Table S4).

SHAP analysis explained model output at global and individual levels (Figure 3 and Supplementary Table S2). The clinical GBDT model was driven primarily by left atrial diameter, triglyceride–glucose index, glucose, uric acid, and smoking history. The radiomic RF model was dominated by epicardial adipose tissue texture and intensity features, particularly ClusterShade, Autocorrelation, and LongRunHighGrayLevelEmphasis. The fusion RF model combined these radiomic signals with glucose, triglyceride–glucose index, and left atrial diameter. These contributions describe predictive behavior and do not establish causality.

## 4 Discussion

In this multicenter study, we developed and externally evaluated clinical, radiomic, and fusion models for AF recurrence after RFA. Radiomic markedly improved discrimination over the clinical feature set. The fusion RF model achieved the highest numerical AUROC, but its advantage over the radiomic RF model was significant only in training and not in either validation cohort. Accordingly, the principal finding is the incremental value of CTA-derived tissue heterogeneity beyond routine clinical variables, rather than definitive superiority of multimodal fusion.

Left atrial structural remodeling remained clinically relevant. Larger left atrial diameter independently predicted recurrence, consistent with chronic stretch, extracellular matrix remodeling, conduction slowing, and heterogeneous refractoriness (24, 25). Left atrial sphericity differed between recurrence groups in the training cohort, but this difference was not consistently reproduced in both validation cohorts. These findings support geometric remodeling as part of the arrhythmogenic substrate while cautioning against treating any single morphological measure as universally dominant (26, 27).

Age, alcohol use, hypertension, diabetes, AF type, NT-proBNP, and left atrial volume were evaluated but did not meet the prespecified training-cohort selection criteria. BMI and left atrial volume index were additionally calculated and likewise did not alter the final feature set. Their non-selection does not negate their established clinical relevance. It may instead reflect the selected population, the limited number of recurrence events, restricted variability after excluding major structural heart disease, and overlap with downstream structural and metabolic markers. Accordingly, retention of left atrial diameter should not be interpreted as evidence of its superiority over left atrial volume index, but as a cohort-specific predictive finding.

Current international guidelines and consensus documents recommend that atrial fibrillation management consists of four core pillars: anticoagulation, rate control, rhythm control, and adjunctive upstream therapy (28). We also conducted research on several therapeutic targets for upstream therapy. In this analysis, metabolic abnormalities and inflammatory activation also appeared to play critical roles in recurrence risk. Our findings further highlight the contribution of metabolic and inflammatory abnormalities to post-ablation recurrence. The triglyceride-glucose index emerged as an important predictor in the clinical model. As a surrogate marker of insulin resistance, the triglyceride-glucose index has been increasingly associated with cardiovascular remodeling, oxidative stress, endothelial dysfunction, and systemic inflammation. Insulin resistance may promote atrial fibrosis by activating inflammatory and profibrotic signaling pathways, including transforming growth factor-*β*–mediated extracellular matrix remodeling (29). Similarly, uric acid level was identified as a significant variable associated with recurrence. Elevated uric acid levels have been linked to oxidative stress, NLRP3 inflammasome activation, calcium-handling abnormalities, and atrial fibrosis, all of which may contribute to electrical and structural remodeling (30). A history of smoking was also associated with recurrence risk, potentially through chronic inflammatory activation, autonomic imbalance, oxidative stress, and collagen deposition (31). Importantly, smoking is a potentially modifiable risk factor, underscoring the importance of lifestyle management and long-term follow-up (32).

Some overlap between the triglyceride-glucose index and glucose is inevitable because glucose is included in its calculation. However, glucose reflects glycaemia at a single measurement, diabetes is a treatment- and duration-dependent diagnosis, BMI reflects general adiposity, and the triglyceride-glucose index integrates lipid and glucose metabolism as a surrogate of insulin resistance. Thus, these variables are related but not interchangeable. The retention of both the triglyceride-glucose index and glucose should be interpreted as shared yet incremental predictive information, not as evidence of independent causal pathways.

Another important finding was the added predictive value of radiomic features. The radiomic model was mainly driven by texture, shape, and first-order features from the left atrium and epicardial adipose tissue. Several GLCM-, GLRLM-, and first-order-derived features contributed consistently across models. These features describe gray-level heterogeneity and spatial distribution but do not directly represent specific biological processes. Previous studies have reported associations between radiomic signatures and fibrosis, inflammation, extracellular matrix remodeling, adipofibrosis, and tissue heterogeneity (33–35). However, whether the features identified in our study reflect these processes remains uncertain. Their biological interpretation should therefore be considered exploratory and hypothesis-generating rather than evidence of a mechanistic relationship (36, 37).

Epicardial adipose tissue radiomic features also contributed to model prediction. Epicardial adipose tissue has been implicated in AF through inflammatory, metabolic, oxidative, and autonomic pathways (38), while its volume and attenuation have been associated with atrial fibrosis and AF recurrence (39). Radiomic may provide additional information beyond conventional volumetric measurements. However, our study did not include histopathological assessment, late gadolinium enhancement MRI, or other tissue-characterisation reference standards. Thus, the biological significance of these features cannot be directly established. Prospective studies incorporating histopathological and advanced imaging validation are required to confirm their underlying biological correlates.

An important strength of our study is the interpretability of the predictive framework. SHAP analyses showed that the clinical model primarily reflected systemic metabolic and structural status, whereas the radiomic model captured spatial heterogeneity and tissue-level microstructural alterations. The fusion model integrated both types of information and achieved the best overall performance, indicating a complementary relationship between clinical and radiomic variables.

Several limitations should be acknowledged. First, this retrospective multicenter study may be vulnerable to selection bias and unmeasured confounding, despite external validation. Although all 877 analyzed patients completed the fixed 12-month outcome assessment, requiring complete follow-up may have introduced complete-case selection bias; because follow-up duration was fixed, a conventional median follow-up was not applicable. Second, ROI delineation and morphological measurements were performed manually, which may have introduced observer-dependent variability. Future studies that incorporate automated segmentation and foundation-model-based workflows may improve reproducibility. Third, histopathological validation and late gadolinium enhancement magnetic resonance imaging data were unavailable; therefore, the biological interpretation of the radiomic signatures remains indirect. Fourth, obstructive sleep apnea, harmonized left ventricular function, longitudinal antiarrhythmic drug exposure, and sufficiently detailed procedural variables were unavailable. Although all patients underwent pulmonary vein isolation, operator-dependent additional ablation may have introduced residual confounding. Fifth, data regarding pharmacological treatments were not available in the present study. Sixth, the sensitivity of follow-up surveillance in this study was limited. The primary endpoint excluded arrhythmias occurring during the prespecified 90-day blanking period and was assessed using the same scheduled Holter protocol across cohorts. Nevertheless, intermittent 24-hour recordings may miss brief or asymptomatic recurrence, and examinations performed outside participating centres could not be fully standardized. The observed recurrence rates may therefore underestimate the true arrhythmia burden. Finally, all participating centers were located in China; international validation is needed to assess generalizability across different ethnic populations and healthcare systems.

In conclusion, we developed and externally validated a multimodal radiomic-based predictive framework for AF recurrence after RFA. By integrating clinical, morphological, and radiomic features, the proposed model achieved strong predictive performance and provided imaging surrogates of inflammatory and fibrotic atrial remodeling. The selected variables should be regarded as cohort-specific predictive markers rather than independent causal determinants or replacements for established electrophysiological predictors. These findings may facilitate precise risk stratification and support individualized management in patients undergoing RFA for AF.

## Data Availability

The original contributions presented in the study are included in the article/Supplementary Material, further inquiries can be directed to the corresponding author/s.
