# Supplementary material for: Integrating preoperative multiregion radiomic features with clinical data to predict atrial fibrillation recurrence after radiofrequency ablation
Source: Front Cardiovasc Med. 2026 Aug 26;13:1923774. doi: 10.3389/fcvm.2026.1923774 (PMC13555556; doi:10.3389/fcvm.2026.1923774)
Supplement: Supplementary file 1 [file Datasheet1.docx]

**Supplemental Online Content**

[eMethods	2](#_Toc236082832)

[Treatment Protocol	2](#_Toc236082833)

[CTA acquisition	2](#_Toc236082834)

[Figure S1.Example of morphological characteristics measurements of the left atrium and pulmonary veins	3](#_Toc236082835)

[Measurement and calculation formulas of morphological parameters related to the left atrium and pulmonary veins	3](#_Toc236082836)

[Figure S2. Examples of left atrium and pulmonary vein regions of interest outlined and the extraction of epicardial fat	4](#_Toc236082837)

[Table S1 Definitions and calculation of CTA-derived left atrial morphological parameters.	5](#_Toc236082838)

[ROI delineation and radiomic feature extraction	9](#_Toc236082839)

[Table S2. Principal predictors in the clinical, radiomics, and fusion models and their potential interpretations.	9](#_Toc236082840)

[Feature Selection, Model Development, and Validation	10](#_Toc236082841)

[1. Dataset Partitioning and Outcome Definition	10](#_Toc236082842)

[2. Clinical Feature Selection	10](#_Toc236082843)

[3. Radiomics Feature Selection and Dimensionality Reduction	11](#_Toc236082844)

[4. Model Framework and Learning Algorithms	12](#_Toc236082845)

[5. Training Strategy and Prediction Generation	13](#_Toc236082846)

[6. Model Evaluation and Selection of Best-Performing Algorithms	13](#_Toc236082847)

[eResults	14](#_Toc236082848)

[Table S3 Baseline characteristics stratified by atrial fibrillation recurrence status.	14](#_Toc236082849)

[**Table S4. Radiomic Models’(RF) Performance Using Siemens Somatom FORCE or GE Revolution CT in All Cohorts.**	15](#_Toc236082850)

[Table S5. Predictive performance of the clinical, radiomics, and fusion models in the training and validation cohorts.	16](#_Toc236082851)

# eMethods

## Treatment Protocol

Radiofrequency Ablation procedure: All patients underwent electrode catheter placement via the left subclavian vein into the coronary sinus under local anesthesia, followed by insertion of two 8F Swartz L1 sheaths through the right femoral vein. After transseptal puncture, the sheaths were advanced into the left atrium. The CARTO system was used to construct an electroanatomical map of the left atrium. Guided by a circular mapping catheter, circumferential isolation of the left and right pulmonary veins was performed sequentially. Substrate mapping was then conducted, and additional linear ablation lesions were created as needed, including at the left atrial isthmus, roof, right atrial isthmus, and between the left and right pulmonary vein isolation circles. In patients with complex fractionated atrial electrograms, targeted ablation of these sites was performed. If sinus rhythm was not restored, electrical cardioversion was administered. After ablation, a 20-minute observation period was undertaken to confirm pulmonary vein isolation and bidirectional block along the ablation lines.

## CTA acquisition

CT scan protocol for the LA and PVs. Pre-examination: Fast 4–6 hours; avoid caffeine/tea polyphenols for 12 hours. Arrive 15 minutes early for IV placement, remove chest metal/accessories, and sign consent. During exam: Supine, arms raised. Scan from aortic arch to cardiac diaphragmatic surface (adjustable). Use non-ECG gating for arrhythmia; ECG gating for regular rhythm. Contrast: Iodine 320–370 mg/ml, rate 5.0 ml/s, total 60 ml contrast + 30 ml saline. Inject 50–60 ml contrast, then 25–40 ml saline. Delay 25–30 s; timing based on peak target vessel enhancement. Scan parameters: Tube voltage CARE kV, rotation 0.28 s, slice 0.75 mm, interval 0.50 mm. Use Siemens Somatom FORCE or GE Revolution CT. Connect ECG leads; perform scout scan. Apply contrast tracking (ROI in ascending aorta). At 100 Hu, delay 6 s for first scan, then 30 s for second. Reconstruction: Multi-phase (0.75 mm slice, 0.50 mm interval), R-R interval at end-diastole. Transmit to Siemens workstation for multiplanar and volume reconstruction.

### Figure S1.Example of morphological characteristics measurements of the left atrium and pulmonary veins

(A)The left atrium diameter and vertebral body diameter at the same level.(B)The maximal diameter and minimal diameter of left atrium.(C)The maximal diameter, minimal diameter and cross section areas of right inferior pulmonary vein.

### Measurement and calculation formulas of morphological parameters related to the left atrium and pulmonary veins

LAV=4／3π（LAD／2）（LA1／2） （LA2／2）

Using the maximum value among LAD, LA1, and LA2 as the approximate maximum left atrial diameter, calculate the diameter of a sphere of the same volume($\text{D=2}\sqrt[\text{3}]{\text{3V/4π }}$).LASP（％）＝Diameter of equal-volume sphere / Maximum diameter of left atrium.^1^

### Figure S2. Examples of left atrium and pulmonary vein regions of interest outlined and the extraction of epicardial fat

CTA, Computed Tomography Angiography.

## Table S1 Definitions and calculation of CTA-derived left atrial morphological parameters.

| Parameter | Full term | Anatomical definition | Plane and direction | Calculation | Unit and interpretation |
| --- | --- | --- | --- | --- | --- |
| LAD | Left atrial diameter | Maximal left-to-right intracavitary diameter of the left atrial body at the level of the right inferior pulmonary vein ostium. | Axial CTA plane through the right inferior pulmonary vein ostium; transverse left-to-right direction. | Direct measurement between opposing endocardial borders. | cm; transverse left atrial size. |
| VD | Vertebral body diameter | Maximal transverse diameter of the vertebral body on the same image as LAD; posterior elements and transverse processes are excluded. | Same axial plane as LAD; transverse direction. | Direct measurement between the lateral cortical margins of the vertebral body. | cm; same-level internal scaling reference. |
| LAVD | Left atrio-vertebral diameter ratio | Ratio of LAD to VD measured on the same axial plane. | Axial plane through the right inferior pulmonary vein ostium. | LAVD = LAD / VD | Dimensionless; atrial transverse size relative to vertebral body width. |
| LA1 | Superior-inferior left atrial diameter | Maximal cranio-caudal dimension of the left atrial body from the atrial roof to the mitral annular plane. | Sagittal multiplanar reconstruction aligned with the left atrial long axis; superior-inferior direction. | Direct measurement. | cm; longitudinal left atrial dimension. |
| LA2 | Anteroposterior left atrial diameter | Maximal anteroposterior dimension of the left atrial body measured perpendicular to LA1. | Same sagittal multiplanar reconstruction; anteroposterior direction. | Direct measurement. | cm; anteroposterior left atrial dimension. |
| Dmax | Maximum left atrial diameter | Largest of the three approximately orthogonal left atrial diameters. | Derived from axial and sagittal measurements. | Dmax = max(LAD, LA1, LA2) | cm; reference diameter for sphericity. |
| LAV | Ellipsoid-estimated left atrial volume | Estimated volume of the left atrial body based on the three measured diameters; pulmonary veins and the left atrial appendage are excluded. | Derived parameter under a triaxial ellipsoid assumption. | LAV = π/6 × LAD × LA1 × LA2 | cm³ or mL; geometric estimate, not segmentation-derived 3D volume. |
| Deq | Equal-volume sphere diameter | Diameter of a theoretical sphere with the same volume as the ellipsoid-estimated LAV. | Derived parameter. | Deq = ∛(6LAV / π) | cm; intermediate quantity for LASP. |
| LASP | Left atrial sphericity | Ratio of the equal-volume sphere diameter to the maximum measured left atrial diameter. | Derived from Deq and Dmax. | LASP = Deq / Dmax; ×100 when reported as % | Dimensionless or %; higher values indicate a more spherical geometry. |

Table note. LAD, LA1, and LA2 describe three approximately orthogonal anatomical directions, although LAD is measured on an axial image and LA1 and LA2 on a sagittal multiplanar reconstruction. LAV is based on a triaxial ellipsoid assumption and is not equivalent to segmentation-derived three-dimensional atrial volume. BSA and LAVI were not added because they were not prespecified and indexing would not remove the geometric limitations of the ellipsoid estimate.

## ROI delineation and radiomic feature extraction

Two radiologists independently delineated the left atrial and epicardial adipose tissue ROIs using 3D Slicer. Fifty examinations were randomly selected for reproducibility assessment, and one radiologist repeated the segmentation after a 4-week interval. Interobserver and intraobserver agreement were evaluated using single-measure, absolute-agreement ICCs based on two-way random-effects and two-way mixed-effects models, respectively. Only features with both ICCs ≥0.80 were retained.

Radiomic features were extracted in three dimensions using PyRadiomics (version 3.0.1). Images and masks were resampled to isotropic 1×1×1-mm³ voxels using B-spline and nearest-neighbour interpolation, respectively. Image-level intensity normalisation was not applied, and CT intensities were discretised using a fixed bin width of 25 HU. Based on the Rad-Z file, three categories of features were extracted from the original images: first-order features describing the intensity distribution; shape features characterising two- and three-dimensional ROI geometry; and texture features derived from GLCM, GLRLM, GLSZM, GLDM, and NGTDM. LoG and wavelet filters were not applied. Extracted features were Z-score standardised using parameters derived exclusively from the training cohort and subsequently applied unchanged to the validation cohorts.

## Table S2. Principal predictors in the clinical, radiomics, and fusion models and their potential interpretations.

The clinical-model section presents all five retained predictors. Odds ratios and 95% confidence intervals were derived from multivariable logistic regression and indicate the direction of association; estimates for continuous variables are reported per 1-standard-deviation increase, whereas smoking history compares patients with versus without a smoking history. The radiomics-model section presents the 12 features retained after LASSO screening and random-forest importance ranking. Positive or negative directions refer to the LASSO coefficient signs and should not be interpreted as necessarily linear effects in the final random-forest model. The fusion-model section presents the six leading SHAP contributors. SHAP values quantify contributions to model output rather than biological causality. Radiomic feature names follow PyRadiomics nomenclature, and their proposed biological interpretations are exploratory because direct histopathological or advanced tissue-characterisation validation was unavailable.

Abbreviations: CI, confidence interval; CTA, computed tomography angiography; EAT, epicardial adipose tissue; GBDT, gradient boosting decision tree; GLCM, gray-level co-occurrence matrix; GLDM, gray-level dependence matrix; GLRLM, gray-level run-length matrix; LASSO, least absolute shrinkage and selection operator; OR, odds ratio; RF, random forest; SHAP, SHapley Additive exPlanations; TyG, triglyceride–glucose index; UA, uric acid.

## Feature Selection, Model Development, and Validation

### 1. Dataset Partitioning and Outcome Definition

The dataset comprised a training cohort (n = 449), an internal validation cohort (n = 192), and an external validation cohort (n = 236). The outcome was recurrence of atrial fibrillation (AF) after radiofrequency ablation and was encoded as a binary variable (recurrence = 1; no recurrence = 0). All feature-selection and model-development procedures were restricted to the training cohort to prevent information leakage. The internal and external validation cohorts were used exclusively to evaluate model performance and generalizability.

### 2. Clinical Feature Selection

A total of 44 candidate clinical variables were considered. These included demographic characteristics, lifestyle factors, comorbidities, laboratory measurements, CHA₂DS₂-VASc and HAS-BLED scores, and morphological measurements of the left atrium and pulmonary veins.

#### 2.1 Univariable Logistic Regression Screening

A separate univariable logistic regression model was fitted for each candidate variable. Continuous variables were standardized to one standard deviation (SD) before analysis; odds ratios (ORs) and 95% confidence intervals (CIs) were therefore expressed per 1-SD increase, allowing comparability across variables measured on different scales. Binary variables were analyzed using their original coding. A two-sided P value < 0.05 was used as the criterion for entry into the multivariable analysis.

Seven variables met this criterion: left atrial diameter, left atrio-vertebral diameter ratio, left atrial sphericity, triglyceride–glucose index (TyG), glucose, uric acid, and smoking history.

#### 2.2 Multivariable Logistic Regression

The seven variables identified in the univariable analysis were evaluated in a multivariable logistic regression model, with consideration of prior evidence and clinical interpretability. Five variables remained statistically significant and were retained for construction of the clinical and fusion models: left atrial diameter, triglyceride–glucose index (TyG), smoking history, glucose, and uric acid (UA).

All five retained variables remained statistically significant in the multivariable model (P < 0.05), and the directions of association were consistent with those observed in the univariable analyses. The clinical feature-selection pathway was therefore 44 candidate variables → 7 univariable associations → 5 multivariable predictors.

### 3. Radiomics Feature Selection and Dimensionality Reduction

To reduce redundancy and multicollinearity in the high-dimensional radiomics feature space and to improve model stability and generalizability, a multistage feature-selection strategy was used. The sequential procedure comprised variance-based filtering, correlation analysis, least absolute shrinkage and selection operator (LASSO) regression, and random forest–based feature-importance ranking.

Variance-based filtering was first applied to remove features with limited variation. Pairwise correlation coefficients were then calculated and a feature-correlation matrix was constructed to further reduce redundancy and multicollinearity. After variance and correlation filtering, LASSO regression was used for additional shrinkage and selection, yielding 78 candidate radiomics features.

A random forest (RF) model was subsequently fitted to the 78 LASSO-selected features to estimate feature importance. Importance was quantified using the mean decrease in impurity (Gini importance), which reflects the contribution of each feature to improving node purity. Features were ranked in descending order of Gini importance. Considering both predictive performance and feature dimensionality, the 12 highest-ranking features were retained as the final radiomics feature subset.

To prevent information leakage and consequent overestimation of model performance, every feature-selection step that depended on the data distribution or outcome—including variance filtering, correlation filtering, LASSO tuning, and RF importance ranking—was performed exclusively within the training cohort. The selection rules and fixed 12-feature subset derived from the training cohort were then applied unchanged to the independent validation cohorts.

### 4. Model Framework and Learning Algorithms

Three model families were developed from the selected feature sets:

Clinical model: the five clinical predictors retained in the multivariable analysis.

Radiomics model: the final 12-feature radiomics subset selected after LASSO and RF ranking.

Fusion model: the five clinical predictors concatenated with the 12 radiomics features, yielding a 17-dimensional input.

Each feature set was evaluated using five supervised learning algorithms: logistic regression (LR), a support vector machine with a radial basis function kernel and probability estimation enabled (SVM), random forest (RF), gradient-boosting decision tree (GBDT), and distance-weighted k-nearest neighbors (KNN).

For LR, SVM, and KNN, features were transformed using RobustScaler before model fitting. In the fusion models, the scaled clinical feature block was multiplied by 0.2 to reduce imbalance in scale and relative contribution between the clinical and radiomics blocks. RF and GBDT were trained using imputed but otherwise unscaled numerical features. Class imbalance was handled using algorithm-appropriate weighting, such as class_weight="balanced" or balanced_subsample. A random seed of 42 was used throughout to support reproducibility.

### 5. Training Strategy and Prediction Generation

All models were fitted using the training cohort. To avoid optimistic bias from evaluating a model on the same observations used for fitting, training performance was estimated from out-of-fold (OOF) predictions generated by five-fold stratified cross-validation. Within each fold, the model was fitted on four training folds and used to generate recurrence probabilities for the held-out fold. The OOF probabilities from all five folds were then pooled to calculate the training AUROC and other performance metrics.

For evaluation in the internal and external validation cohorts, each final model was refitted using the complete training cohort and then applied to the corresponding validation cohort. The resulting patient-level recurrence probabilities were used for receiver operating characteristic analysis, calibration assessment, decision curve analysis (DCA), DeLong testing, and SHapley Additive exPlanations (SHAP) analysis.

### 6. Model Evaluation and Selection of Best-Performing Algorithms

The primary measure of discrimination was the area under the receiver operating characteristic curve (AUROC). Ninety-five percent CIs were estimated using 1,000 bootstrap resamples. At the threshold maximizing the Youden index, sensitivity, specificity, precision, F1 score, and accuracy were also reported.

Calibration was evaluated using calibration curves and the Brier score. Clinical net benefit was assessed by decision curve analysis. Pairwise differences in AUROC between models were evaluated using the paired DeLong test.

Based on performance in the external validation cohort, the best-performing algorithms were GBDT for the clinical model, RF for the radiomics model, and RF for the fusion model. These models were further examined using SHAP beeswarm, waterfall, and heatmap plots to characterize global and individual-level feature contributions to model output.

**eResults**

**Table S3 Baseline characteristics stratified by atrial fibrillation recurrence status.**

Baseline characteristics are presented for the training, internal validation, and external validation cohorts and stratified by recurrence status. Continuous variables are expressed as median (interquartile range), and categorical variables as n (%). P values represent within-cohort comparisons between the recurrence and non-recurrence groups. Comparisons in the validation cohorts were descriptive and were not used for feature selection or model optimization. Recurrence was defined as documented atrial fibrillation, atrial flutter, or atrial tachycardia lasting >30 s after the 90-day blanking period and within 12 months after radiofrequency ablation.

Abbreviations: AF, atrial fibrillation; Hb, hemoglobin; HDL, high-density lipoprotein; HR, heart rate; LDL, low-density lipoprotein; NT-proBNP, N-terminal pro-B-type natriuretic peptide; Scr, serum creatinine; TC, total cholesterol; TG, triglyceride; TyG, triglyceride–glucose index; UA, uric acid.

**Table S4. Radiomic Models’(RF) Performance Using Siemens Somatom FORCE or GE Revolution CT in All Cohorts.**

| Study Cohorts | AUROCs | 95%CI |
| --- | --- | --- |
| Training cohort | | |
| Siemens Somatom FORCE CT (N=285) | 0.930 | 0.892-0.961 |
| GE Revolution CT (N=164) | 0.908 | 0.854-0.948 |
| Internal validation cohort | | |
| Siemens Somatom FORCE CT (N=112) | 0.845 | 0.754–0.916 |
| GE Revolution CT (N=80) | 0.826 | 0.704–0.913 |
| External validation cohort | | |
| Siemens Somatom FORCE CT (N=94) | 0.829 | 0.742–0.896 |
| GE Revolution CT (N=142) | 0.842 | 0.778–0.891 |

**Table S5. Predictive performance of the clinical, radiomics, and fusion models in the training and validation cohorts.**

Performance is reported for five algorithms applied to the clinical, radiomics, and fusion feature sets in the training, internal validation, and external validation cohorts. The clinical feature set comprised left atrial diameter, triglyceride–glucose index, smoking history, glucose, and uric acid; the radiomics feature set comprised 12 selected features; and the fusion model combined all 17 features. Training performance was estimated from pooled out-of-fold predictions generated by five-fold stratified cross-validation. Validation predictions were generated after refitting each model on the complete training cohort. AUCs are presented with 95% confidence intervals estimated using 1,000 bootstrap resamples. Threshold-dependent metrics were calculated at the corresponding threshold maximizing the Youden index. Prevalence denotes the proportion of patients with recurrence.

Abbreviations: AUC, area under the receiver operating characteristic curve; CI, confidence interval; FN, false negative; FP, false positive; GBDT, gradient boosting decision tree; KNN, k-nearest neighbors; LR, logistic regression; NPV, negative predictive value; RF, random forest; SVM, support vector machine; TN, true negative; TP, true positive.

**References**

[1]	Moon J, Lee HJ, Yu J, et al. Prognostic implication of left atrial sphericity in atrial fibrillation patients undergoing radiofrequency catheter ablation. Pacing and clinical electrophysiology : PACE. 2017;40:713-720.
